# Practical Medicine

**Published:** 1877-01

**Authors:** 


### II. Practical Medicine.

1. *Disorders occurring to a Laborer in a Manufactory of Aniline.* Fredet. (*L' Union Médicale*, No. 119.)

A man, thirty-seven years old, was employed in a laboratory where he inhaled the vapors of nitro-benzine and aniline. Before engaging in this work he had enjoyed perfect health. In the course of nineteen months he suffered from vertigo, malaise and vomiting, for which a purgative was ordered—the routine treatment among artizans of this manufactory, as soon as the sclerotic assumes a yellow hue and the gums become blue. The hours of labor were from 6 A. M. to 6 P. M., with necessary intervals for meals.

Upon admission to hospital he was found to be pale, his tissues puffy, the sclerotic of each eye subicteroid in color, gums blue without ulceration, numerous purpuric blotches on the legs, and pustules of ecthyma upon the internal faces and upper portions of the thighs. There was also œdema and a furuncular eruption over the malleoli, eczema, hemeralopia, general feebleness and difficulty in progression. A bruit was audible over the large vessels of the neck, simultaneous with the first cardiac sound. Pulse feeble and slow; no albumen in the urine; saburral condition of the primæ viæ; frequent stools and thirst.

These phenomena are due to the action of more than one poison — nitro-benzine, aniline and arsenic. At first there was vertigo, malaise, etc., due to the vapor of nitro-benzine; then followed the gastric disturbance and nervous depression, due to the aniline; finally, the furuncular and pustular eruption, with the eczematous affection, arose in consequence of subjection to the arsenical influence.

2. *Chylous Urine.* Robin. (*Le Progrès Méd.*, November 25, 1876.)

Robin exhibited to the Anatomical Society the chylous urine voided by a lady, each morning only, for three years. Decubitus consequently seemed to have some influence upon its production. The microscope showed cellular *débris*, delicate granules having amœboid movements, but no drops of fat. The urine contained no albumen, no sugar, and but one-third of the normal proportion of urea.

In the discussion of the specimen which followed, it was asked whether hæmaturia had previously existed. Crevaux having shown that in warm countries chyluria occasionally alternated with hæmaturia, Renant remarked that in chylous urine there was in general a distinct diapedesis of red and white blood globules, but in an abnormal proportion. Parasites were also occasionally discovered. The physicians practicing in warm latitudes have noticed, besides, varices of the lymphatics of the groin and scrotum, and even elephantiasis; and these lymphatic alterations have been ascribed to a parasite of the genus *filaria*.

Robin remarked that the saline constituents of this specimen of urine seemed closely analogous to those of chyle.

3. *Regeneration of Nerves after Neurotomy.* MITCHELL AND BERTOLET. (*Amer. Jr. M. Sc.*, April, 1876.)

From an injury in the median territory of the right palm of a Miss T., there occurred intense neuralgia, which no treatment improved. In November, 1871, Dr. Sapolini "cut down on the musculo-spiral nerve" "and removed one inch of it. This operation eased the neuralgia, which, however, came back in eight days suddenly." Atrophy of the extensor group of the forearm, with loss of power to extend the wrist, and in the common extensor of the fingers, resulted. The loss of sensation in the hand was small. In six months the wasted muscle began to enlarge again and the power, to return. In February, 1873, she could partially extend the hand, and "there was normal touch in all the radial region in the hand." In March, 1873, Dr. Mitchell had Dr. Brinton (J. H.) cut down and remove three-fourths of an inch of the median nerve in the forearm of this patient, in the hope of stopping her suffering. "The lower end of the cut nerve was doubled upon itself, and secured by a thread, to prevent reunion," and "the interval betwixt the nerve ends became by measure two and a half inches." In January, 1874, pain began in the line of the incision over the musculo-spiral nerve, and soon excessive pain in the radial region of the back of the hand. Swelling pain and redness occurred, and an abscess was looked for, but it did not occur. To relieve the constant torture of the patient, and fully believing the cut nerve had reunited, Dr. M. had Dr. B. cut down through the old incision and again remove a portion of the nerve. The nerve was found to have become re-united, two small swellings indicating the site of the two ends of the cut nerve. The operation was soon followed by perfect relief, which a year later had continued. Dr. M. thinks this case is the first one recorded "in which a nerve once cut has after repair been cut a second time, so as to remove the restored portion." Here nerve repair must have occurred within six months after the operation, as shown by the restoration of the wasted muscles. "Before we cut the median, and since, I frequently faradized the musculo-spiral nerve above the section, and threw the extensors into play." During the second operation, and when the nerve was laid bare, he repeated the test by putting the conductors on the exposed nerve itself.

Dr. M. thinks this case further proves the theory which he holds to, that if a single nerve be left uncut in a member, some sensitiveness will remain over the whole of the part, which will gradually improve. He believes this gradual return of power to occur through the coarse and fine anastomosis of the nerve fibers. This power "comes slowly, and we learn by degrees to use the new channels." In the case under consideration this gradual increase in sensitiveness was a marked peculiarity, while the paralysis in many muscles of the hand was complete and permanent after the two operations of Dr. B.

Two cases are quoted from Dr. Hodge where cicatrices of former nerve sections were cut down upon, and the nerves found re-united. In each instance, too, there was the enlargement at the site of the cut end of the nerve. In one of these cases an inch, and in the other two inches, of nerve was excised.

In Dr. M.'s case "microscopical examination showed the central enlargement to consist almost entirely of double contoured nerve fibers, which interlace and cross one another in every possible direction." There was a moderately increased quantity of connective tissue. The distal enlargement presented the same histological features. "Horizontal and vertical sections of the intermediate portions of the nerve, between the neuromatous swellings, are indistinguishable from those obtained from a perfectly normal nerve; thus showing how complete has been the regeneration of the excised part."

The cicatricial neuromata in this case resemble those of the lower animals under similar circumstances; there, as here, the central swelling is always the larger. Dr. B. believes the evident increase by segmentation in the nuclei of the neurilemma in this case, "leaves little room for doubt that also in the human subject these nuclei are the principal factors in the reproduction of divided nerves." He has experimented upon several kittens, dividing the ischiatic nerves and watching the process of repair. The results are confimatory of the observations of others. The power of regeneration is stronger the younger the animal.

The first traces of regeneration show themselves a few days after the ligation of the nerve. The commencement is due to the active proliferation of the nuclei of the neurilemma. "They multiply by segmentation, are arranged in rows and surrounded by a layer of protoplasm; have roundish, oval and even spindle forms. This proliferation is seen in the central swelling and throughout (Benecki) the peripheral end of the nerve; the increased nuclei elongate into long spindles, the medullary substance disappearing. These spindle-shaped nuclei send out long filamentous protoplasmic processes, which unite with one another and widen into narrow bands. These fibers are next surrounded by a second 'glittering contour.' These short cylinders gradually blend into one continuous medullary sheath. The nerve sheath being next filled out at all points with medullary substance around the axis-cylinder, present a uniform width throughout, and the regenerative process is now complete."

"The reunion of the separate nerve ends, in cases of excision, does not take place by the primitive fibers of the central and peripheral ends simply growing toward each other," "but is brought about by the coalescence of spindle cells, arranged in rows in the intermediate cicatricial portion, which, at the same time, unite with the primitive fibers of both ends."

